# Indents

**Published:** 1907-03-15

**Authors:** 


### INDENTS.

**Brief Articles of Technical or Scientific Value will receive notice and comment. Contributions invited.**

**Solution to Remove Stains of Silver Nitrate and Other Salts Sensitive Dentin.** Ammonium Chloride, 1 part; Mercury Bichloride, 1 part; water, 8 parts. Apply and wash off with soap and water.—W.H Tweedle, in December *Review.*

**Deodorizer.** Tr. Lavender Comp., costs sixty cents per pound and may be diluted to make a gallon or more. A few drops of the diluted solution in your cuspidor will have a pleasant effect on your patients—H. E. Davis, *Dental Review.*

**Fragrant Mouth Wash.** Two drops of camphor on your tooth brush will give your mouth its freshest, cleanest feeling imaginable, will make your gums rosy, and absolutely prevent anything like cold-roses or affections of the tongue. *Dental Digest.*

**Retention of Lower Dentures.** About five years ago a lady called at my office to have me construct for her a lower denture. Upon examination I found that the alveolus from the region of the first bicuspid on either side was almost completely obliterated, and in the region of the anterior teeth there was a flat ridge, rising about one-fourth of an inch above the attachment of the buccal and lingual muscles.

I took great pains in constructing the denture after the ordinary method, paying particular attention to the articulation, and making the teeth a little shorter than they really should have been for appearance sake, to reduce the leverage as much as possible, and sent my patient home with instructions to exercise patience and perseverance for about two weeks, and if she were not getting satisfaction to return. I might say that I really expected her to return. When she did return, she complained of the denture sliding from one side of the mouth to the other.

I again examined the mouth, and thought that it might be possible to extend the inner flange of the denture back inside the ramus to near the anterior pillar of the fauces. I then took an ordinary impression tray and some wax and made the extensions on the tray. I took the impression and to my satisfaction found that I had a splendid impression of the process and the inner part of the ramus, and that the patient experienced no more unpleasantness than in the ordinary way. I constructed the denture from this impression, and found that the patient could wear it with much comfort. Consequently I have been using this method ever since when I met a case with a poor process.

I might say that I have noticed that where the alveolus is badly absorbed, it is much easier to get an impression of the inner part of the ramus. This method not only prevents the denture from moving from side to side, but it also prevents a backward motion; in fact, it places the denture, as it were, within a dovetail space.

Having constructed a denture after this manner, it is still all-important to see that the articulation is perfect; because a lower denture has not so much retention, even with this method. as has an upper. Therefore it is always the weakest and will be the first to move.—A. T. Morrow, *Dominion Dental Journal.*

**The Taggart Inlay Process.** The Taggart inlay differs from all others in two essential particulars. It is made of pure gold, and no preliminary matrix is needed. Another departure from previous methods lies in the fact that neither impression of the cavity nor model is required though in an extreme case such preliminary model may prove a convenience.

The Taggart method is as follows: A specially prepared wax is utilized. The exact formula for this wax has not as yet been given, but is it, in the main, a combination of wax and paraffine, which is refined by straining twice through a filtering paper. This precaution removes all foreign particles and renders the material absolutely meltable.

With this special wax the original model of the inlay is constructed. In plainer terms, Dr. Taggart fills the cavity with wax, making his wax conform exactly to the shape required of the finished gold inlay. Where occlusal surfaces are involved, the wax is forced into the cavity, and the patient told to close his mouth, thus assuring the proper "bite." All excess of wax is then carefully cut away, and the remainder carved into perfect occlusal and approximal contour and relation. The approximal surface is perfected by polishing with thin tape and vaseline. In a great degree the final success of the inlay depends upon the care and skill expended upon the wax, because so accurate is Dr. Taggart's casting method that the gold assumes precisely the form of the wax, be it good or bad.

When the wax inlay, so to speak, is perfected, a sprue former, a small bar of brass about the size of No. 16 wire, is attached at a convenient point to the wax, by slightly heating it. Thus the wax is made to adhere to one end of this sprue former, while the other end is set into a hole in the cover of his casting flask. The next step is the investment. The investment is made with Peck's investing material, though other finely ground investments will serve. The powder must be mixed so as to avoid bubbles. It is consequently slowly sifted into the water until the proper consistence is obtained. The wax is covered with the investment very carefully, the tiniest spatula serving to carry just a little of the material to place at a time, after which it is coaxed all around the wax until the latter is completely hidden in a ball-like mass. This is then put aside for a moment to slightly set. The ring of the flask proper is then placed over this invested wax, which rests, be it remembered on the sprue former which is set in the flask cover. The ring in place, more investment is poured into it, thus filling the flask.

When the investment has sufficiently set, the flask is placed over a Bunsen flame for further drying and heating up, with the result that the wax is melted, passing into the investment. Thus we have a disappearing model. The melting of the wax likewise enables the operator to remove the flask cover which brings with it the sprue former which had been attached to the wax; and the withdrawal of this bar leaves a hole leading down to the mold of the inlay which is now empty, the wax having been absorbed by the plaster investment.

The cover of the flask is also so fashioned that its removal leaves a cup-shaped depression in the investment at the centre of which is the hole which leads to the mold. Thus a crucible is supplied into which an ingot of gold may be placed. The flask with this lump of gold is then placed in the casting apparatus, and the flame of an oxy-hydrogen blow-pipe turned directly upon the ingot, playing from above downward. The gold is watched until it is not merely melted, but actually boiling, at which temperature it is as fluid as water. At this precise moment a lever is pushed down which instantly accomplishes three things; the blow pipe arm and flame is turned to one side; the flask is closed with a cover lined with asbestos; and finally thirty pounds of compressed air is brought against the molten mass forcing it into the matrix of the investment, thus making the cast under this heavy pressure. The entire procedure, as above described, occupied Dr. Taggart, at his clinic, only twenty-nine minutes.

The inlays resulting from this method of casting are marvelous. The adaptation is so accurate that there is no doubt but that Dr. Taggart has found a means by which the shrinkage of the metal is entirely overcome. Of course, the shrinkage of high grade metals is not great, but by this process alloyed golds and even base metals may be cast with equal accuracy.—*Items of Interest.*

